# Neurofilament light chain in blood as a diagnostic and predictive biomarker for multiple sclerosis: A systematic review and meta-analysis

**DOI:** 10.1371/journal.pone.0274565

**Published:** 2022-09-14

**Authors:** Liangxia Ning, Bin Wang

**Affiliations:** Department of Neurology, Yuncheng Central Hospital, The Eighth Shanxi Medical University, Yuncheng, China; North Karelia Central Hospital, FINLAND

## Abstract

**Background:**

Neurofilament light chain (NfL) in cerebrospinal fluid (CSF) is a biomarker of multiple sclerosis (MS). However, CSF sampling is invasive and has limited the clinical application. With the development of highly sensitive single-molecule assay, the accurate quantification of the very low NfL levels in blood become feasible. As evidence being accumulated, we performed a meta-analysis to evaluate the diagnostic and predictive value of blood NfL in MS patients.

**Methods:**

We performed literature search on PubMed, EMBASE, Web of Science and Cochrane Library from inception to May 31, 2022. The blood NfL differences between MS *vs*. controls, MS *vs*. clinically isolated syndrome (CIS), progressive MS (PMS) *vs*. relapsing-remitting MS (RRMS), and MS in relapse *vs*. MS in remission were estimated by standard mean difference (SMD) and corresponding 95% confidence interval (CI). Pooled hazard ratio (HR) and 95%CI were calculated to predict time to reach Expanded Disability Status Scale (EDSS) score≥4.0 and to relapse.

**Results:**

A total of 28 studies comprising 6545 MS patients and 2477 controls were eligible for meta-analysis of diagnosis value, and 5 studies with 4444 patients were synthesized in analysis of predictive value. Blood NfL levels were significantly higher in MS patients *vs*. age-matched controls (SMD = 0.64, 95%CI 0.44–0.85, P<0.001), vs. non-matched controls (SMD = 0.76, 95%CI 0.56–0.96, P<0.001) and *vs*. CIS patients (SMD = 0.30, 95%CI 0.18–0.42, P<0.001), in PMS *vs*. RRMS (SMD = 0.56, 95%CI 0.27–0.85, P<0.001), and in relapsed patients *vs*. remitted patients (SMD = 0.54, 95%CI 0.16–0.92, P = 0.005). Patients with high blood NfL levels had shorter time to reach EDSS score≥4.0 (HR = 2.36, 95%CI 1.32–4.21, P = 0.004) but similar time to relapse (HR = 1.32, 95%CI 0.90–1.93, P = 0.155) compared to those with low NfL levels.

**Conclusion:**

As far as we know, this is the first meta-analysis evaluating the diagnosis and predictive value of blood NfL in MS. The present study indicates blood NfL may be a useful biomarker in diagnosing MS, distinguishing MS subtypes and predicting disease worsening in the future.

## Introduction

Multiple sclerosis (MS) is a chronic inflammatory neurodegenerative disease affecting over two million people around the world [1]. The clinical courses and manifestations of MS are highly variable encompassing mild or benign forms that may not need treatment and progressive stage that develops irreversible clinical and cognitive deficits with limited response to standard treatment [2]. Highly effective treatments have been developed and become widely available in recent years [3]. Reliable markers for disease detection, staging and prognosis prediction are warranted for the decision-making of best therapy to improve prognosis.

Neurofilament light chain (NfL) in cerebrospinal fluid (CSF) is an emerging biomarker for MS. NfL is a subunit of neurofilaments constituting neuronal and axonal cytoskeleton in central nervous system (CNS) as well as part of the peripheral nervous system, which is released to CSF and blood when neuronal and axonal damage occur [4]. It directly reflects the neuroaxonal injury in many inflammatory, neurodegenerative, traumatic and ischemic diseases of CNS [5, 6]. Previous studies have found more abundant CSF NfL in MS patients than in sex- and age-matched controls and suggested that CSF NfL may help distinguish MS subtypes [7]. It has also reported as a biomarker for frontotemporal dementia (FTD), Alzheimer’s disease (AD), amyotrophic lateral sclerosis (ALS), and atypical parkinsonian disorder (APD) [8]. However, CSF acquisition is a relatively invasive procedure that limits the clinical application, especially longitudinal and repetitive sampling for disease monitoring, of CSF NfL.

In patients with neurological disorders, NfL is released in a large amount to CSF when neural cells are damaged and eventually into the bloodstream [9]. Previous studies mostly focused on CSF levels since the conventional detection methods, such as enzyme‐linked immunosorbent assay (ELISA) and electrochemiluminescence (ECL)‐based assay, had low sensitivity in quantifying the low blood levels [10, 11]. Recently, the development of highly sensitive single-molecule assay (SIMOA) has allowed the accurate quantification of low blood concentrations of NfL and now been widely used [12]. The blood levels of NfL by SIMOA are nearly 40-fold lower than CSF levels but highly correlated with CSF levels, magnetic resonance imaging (MRI) lesions and clinical symptoms [13, 14]. Serum NfL is now widely accepted to monitor disease activity and response to disease-modifying therapy (DMT) [14, 15], and becomes more and more refined as a biomarker in MS [16].

With the increasing evidence of blood NfL measurements in MS patients, we performed the present systematic review and meta-analysis to evaluate the value of blood NfL in diagnosing MS, distinguishing MS subtypes and severity, and predicting disease worsening.

## Methods

### Literature search strategy

The present systematic review and meta-analysis was performed according to the Preferred Reporting Items for Systematic Reviews and Meta-Analysis (PRISMA) statement [17]. Candidate articles investigating the diagnostic or predictive value of blood NfL levels in MS were systematically searched in electronic literature databases including PubMed, EMBASE, Web of Science and Cochrane Library from inception to May 31, 2022. The following keywords were used for literature search: (“neurofilament light chain” OR “neurofilament-light chain” OR “neurofilament” OR NfL OR sNfL OR pNfL) AND “multiple sclerosis”. Additional relevant articles were obtained by manually searching the reference lists of eligible studies.

### Inclusion and exclusion criteria

All eligible studies should meet the following criteria: (1) measured serum or plasma NfL concentrations in adult MS patients; (2) investigated the diagnostic or predictive value of blood NfL levels; (3) provided sufficient data for meta-analysis. MS was diagnosed according to Poser [18] or McDonald criteria [19–21]. NfL was measured by SIMOA, electrochemiluminescence method (ECL) or enzyme linked immunosorbent assay (ELISA). In details, for diagnostic value, the blood NfL levels were compared between MS vs. controls which included healthy control (HC) and non-inflammatory neurological disease control (NINDC), MS vs. clinically isolated syndrome (CIS), relapsing-remitting MS (RRMS) vs. progressive MS (PMS), and MS in relapse vs. MS in remission. The mean value and standard deviation (SD) of blood NfL levels, or the other statistics that can be converted to mean and SD, in both groups should be provided. For predictive value, hazard ratio (HR) estimate and corresponding 95%CI for high blood NfL levels predicting the time to Expanded Disability Status Scale (EDSS) score ≥4.0 or relapse should be provided. Cases series, meeting abstracts, reviews, meta-analyses and studies with pediatrics patients were excluded. For articles with overlapped samples, only the one with largest sample size was included.

### Quality assessment

For studies comparing the blood NfL levels in two group, the quality was assessed by using Newcastle-Ottawa scale (NOS) for case-control studies, which comprised selection, comparability and exposure domains. For studies investigating the predictive value, the quality was assessed by using NOS for cohort studies, which contained selection, comparability and outcome domains. The total stars assigned to all items were 9. Studies with 5 or 6 stars were considered as moderate-quality studies and those with 7 or more stars were of high quality.

### Data extraction

We extracted the following information from all eligible studies: first author, year of publication, country, diagnostic criteria of MS, sample source (serum or plasma), method of blood NfL measurement, baseline characteristics (sample size, age, gender, EDSS score, disease duration, DMT use). For diagnostic value, the mean value and SD of NfL levels in both groups were extracted. If the studies only provided median value with interquartile (IQR) or range of NfL levels, we converted these values to mean and SD statistics by using methods introduced by Wan *et al* [22] and Luo *et al* [23]. Similarly, the median with IQR or range of baseline age, EDSS score and disease duration were converted to mean with SD when we performed meta-regression analysis. For predictive value, the cutoffs of high NfL levels and the HR estimates for EDSS score≥4.0 or relapse were extracted.

The literature search and selection, quality assessment and data extraction were performed by two independent researchers. Discrepancies were resolved by further discussion of these two researchers.

### Statistical analysis

Between-study heterogeneity was evaluated by I^2^ statistic and Q test. I^2^ <25%, between 25% and 50%, and >50% indicated low, medium and high levels of heterogeneity, respectively. For meta-analysis with I^2^<50% and P value of Q test>0.10, the fixed-effect model was used; otherwise, the random-effect model was applied. The effect sizes were estimated by standard mean difference (SMD) and 95%CI with Cohen’s d [24] for diagnostic value and calculated by HR and 95%CI for predictive value. We considered the SMD of ≤0.2, between 0.2 and 0.8, and ≥0.8 as small, moderate and large effect size, respectively [24]. For MS vs. Control, subgroup analyses regarding control type (HC, NINDC), sample source (serum, plasma), NfL detection method (SIMOA, ECL or ELISA) and DMT use (no, mixed or missing)were performed. Specifically, only if the authors declared enrollment of age-matched controls, the study was classified as age-matched; otherwise it was not age-matched, even though there was no statistical difference by baseline age comparison. Since studies have shown blood NfL was highly correlated with age, we analyzed age-matched studies and non-age-matched studies separately. Meta-regression analyses for mean age, percent of female, mean disease duration, mean EDSS score and sample size were also performed to identify potential source of heterogeneity for meta-analyses including 10 or more eligible studies. Sensitivity analysis was also performed with Leave-One-Out method, i.e. omitting one study and recalculating the pooled effect size each time. Publication bias was assessed by viewing the symmetry of funnel plot and by Egger’s test. All analyses were performed by using STATA 16 (StataCorp, TX, USA).

## Results

### Baseline characteristics of eligible studies

**Fig 1:**
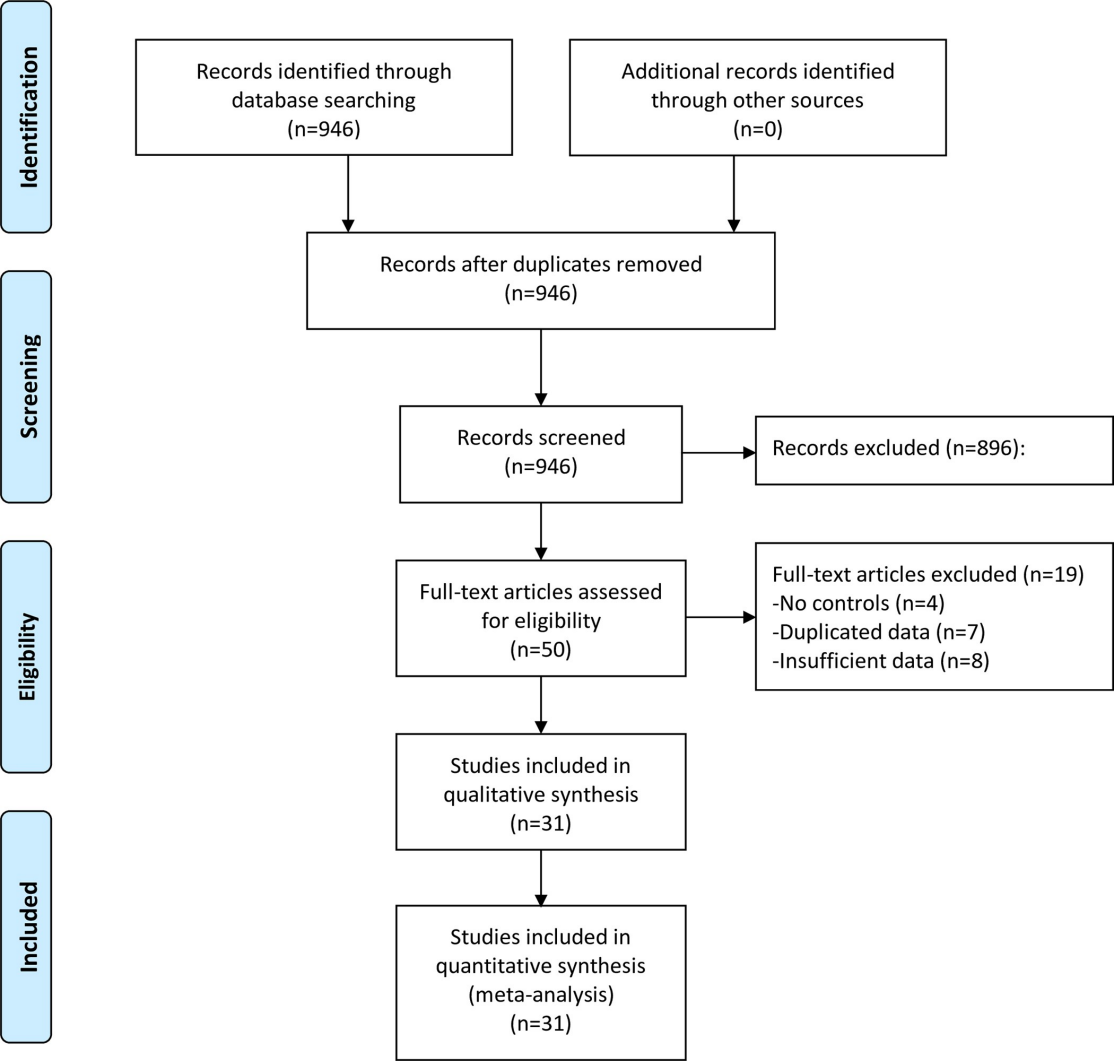
Flowchart of literature search.

A total of 31 studies fulfilling the inclusion and exclusion criteria were finally included in quantitative analysis (Fig 1) [10, 11, 13, 14, 25–51]. Among them, 28 studies comprising 6545 MS patients and 2477 controls were eligible for meta-analysis of diagnosis value (Table 1), and 5 studies with 4444 MS patients were synthesized in meta-analysis of predictive value (S1 Table).

**Table 1:** Characteristics of studies included in meta-analysis for diagnosis value of blood NfL concentrations.

Author	Year	Country	Patient group							Control group				Comparison
			Diagnosis	N	Age, y	%Female	Disease duration, y	EDSS score	DMT use (%)	Condition	N	Age, y	%Female	
Disanto	2015	Various	MS	100	31.2	67	NA	2.18	NA	HC	92	36.4	63	MS vs. HC, MS vs. CIS
Kuhle	2016	Switzerland	MS	31	31.6	64.5	1.32	2	0	HC	18	30.8	55.6	MS vs. HC, Relapse vs. Remission
Disanto	2017	Switzerland	MS, SMSC cohort	246	42.4	65.9	8.21	2.82	50.8	HC	254	44.4	68.1	MS vs. HC
			MS, LUGANO cohort	142	38.5	64.9	NA	NA	NA					
Piehl	2017	Sweden	MS	39	39.6	61.5	NA	2.4	NA	NINDC	27	35.2	55.6	MS vs. NINDC
Novakova	2017	Sweden	PMS	82	48	54.9	NA	5.4	NA	HC	42	28	40.5	PMS vs. RRMS, Relapse vs. Remission
			RRMS	204	40.2	70.1	NA	2.6	NA					
Barro	2018	Switzerland	MS	257	44.5	69.6	11.05	3	64.6	HC	258	44.3	68.6	MS vs. HC, PMS vs. RRMS
Hakansson	2018	Sweden	MS	41	30.29	78	11.8	1.68	0	HC	22	33.1	77.3	MS vs. HC
Abdelhak	2018	Germany	MS in relapse	18	31.8	NA	0.62	1.82	11.1	NA	NA	NA	NA	Relapse vs. Remission
			MS in remission	24	37.4	NA	4.19	2.88	16.7					
Hogel	2018	Finland	MS	79	50.2	70.9	15.48	3.7	64.6	HC	13	47	69.2	MS vs. HC, PMS vs. RRMS
Ferraro	2019	Italy	PMS	70	58.9	30	20	6.32	0	HC	10	56.9	40	PMS vs. RRMS
			RRMS	21	42.9	28.6	9.56	1.32	0					
Watanabe	2019	Japan	MS	49	39	73.5	8.16	4.03	55.1	HC	49	46.2	85.7	MS vs. HC, PMS vs. RRMS
Thebault	2019	Canada	MS	23	27	51.9	7.42	4.82	100	NINDC	33	37.5	72.7	MS vs. NINDC
Jakimovski	2019	US	MS	127	48.4	70.1	16.3	3.2	78.7	HC	52	43.8	86.8	MS vs. HC, MS vs. CIS, PMS vs. RRMS
Sejbaek	2019	Denmark	MS	52	34.1	86.5	NA	1.77	0	HC	23	38.2	87	MS vs. HC
Baldassari	2019	US	MS	22	46.4	68.2	12.4	5.5	0	HC	10	47.1	60	MS vs. HC
Manouchehrinia	2020	Sweden	MS	3092	38.4	70.3	4.23	NA	NA	HC	1026	39.8	73.2	MS vs. HC
Bittner	2020	Germany	MS	445	32.4	67.2	2	1.5	0	NA	NA	NA	NA	MS vs. CIS
			CIS	369	33.4	69.4	0.14	1.5	0					
Thebault	2020	Canada	MS	67	38	70.1	NA	1.5	3.0	NINDC	37	38	81.1	MS vs. NINDC, Relapse vs. Remission
Ayrignac	2020	France	PMS	18	50.8	77.8	3.5	3.86	0	NA	NA	NA	NA	PMS vs. RRMS, Relapse vs. Remission
			RRMS	111	39.9	74.8	7.17	1.35	48.7					
Huss	2020	Germany	PMS	39	53	53.8	NA	5.65	7.7	NA	NA	NA	NA	PMS vs. RRMS
			RRMS	47	36.1	61.7	NA	2.53	14.9					
Olsson	2020	Denmark	MS, cohort 1	49	36.1	65.3	2.94	1.68	0	HC	58	38.1	48.3	MS vs. HC
			MS, cohort 2	68	35.3	76.5	1.18	2	0	HC	50	33	68	MS vs. HC
Bridel	2020	Netherlands	MS	89	45.1	71.9	NA	NA	23.6	HC	88	44.5	44.3	MS vs. HC, PMS vs. RRMS
Saraste	2020	Finland	MS	79	48.1	75.9	14.27	2.91	68.4	HC	10	48.3	70	MS vs. HC, PMS vs. RRMS
Szilasiova	2021	Slovak	MS	159	40.4	64.8	7.54	3.93	100	HC	66	42.5	68.2	MS vs. HC
Liu	2021	China	MS	98	32.1	67.3	5.35	2.18	77.6	HC	84	29.4	64.3	MS vs. HC, Relapse vs. Remission
Cruz-Gomez	2021	Spain	MS	35	38.4	57.1	3.13	1	94.3	HC	23	35.4	56.5	MS vs. HC
Niiranen	2021	Finland	MS	63	49.7	73	21.12	2.06	74.6	HC	14	47.2	50	MS vs. HC
Harp	2022	America	MS	90	37.0	67.8	NA	NA	16.7	HC	118	42.5	60.2	MS vs. HC

NfL: neurofilament light chain; MS: multiple sclerosis; PMS: progressive MS; RRMS: relapsing-remitting MS; CIS: clinically isolated syndrome; HC: healthy control; NINDC: non-inflammatory neurological disease control; EDSS: Expanded Disability Status Scale; DMT: disease-modifying therapy; NA: not available.

For diagnosis value analysis, 4 studies detected plasma NfL (pNfL) concentrations [26, 34, 36, 43] and the others measured serum NfL (sNfL) levels. Two studies applied ECL method [10, 11], one used ELISA [40], and the others adopted the highly sensitive SIMOA mothed for NfL measurements in blood. Ten studies enrolled age-matched controls with MS patients [13, 28, 32, 34–37, 40, 44, 45] and 7 recruited sex-matched controls [28, 32, 34–36, 40, 43]. The other 18 studies that did not declare whether controls were age-matched were then considered as not age-matched studies, even though there was no statistical difference of age at baseline comparison in some studies. As to DMT use, 7 recruited treatment-naïve patients [10, 28, 32, 35, 36, 42, 46], while the other studies reported a proportion of patients treated with DMT or missing information of DMT use. Quality assessment using NOS for case-control studies identified 19 moderate-quality studies that had 5 or 6 stars and 9 high-quality studies with 7–9 stars (S2 Table). The characteristics of the included studies were summarized in Table 1.

Among studies exploring the predictive value of blood NfL concentrations, 3 measured sNfL and 2 detected pNfL [32, 34, 41, 47, 48]. The cutoffs for high NfL levels were 80th percentile of age-corrected reference values in two studies but differed in the other studies. Two studies investigated the association of high blood NfL level with time to relapse and 3 with time to reaching ESS score≥4.0. All studies were awarded with 7 stars according to NOS for cohort studies (S3 Table). The characteristics of these studies were summarized in S1 Table.

### MS vs. control

**Fig 2:**
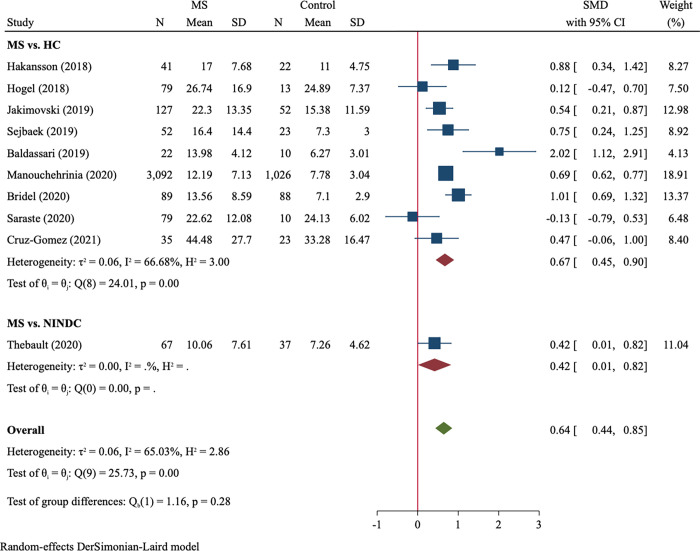
Forest plot of blood NfL concentrations between MS patients *vs*. age-matched controls. NfL: neurofilament light chain; MS: multiple sclerosis; HC: healthy control; NINDC: non-inflammatory neurological disease control; SMD: standard mean difference.

Twenty-three studies compared blood NfL between MS patients and controls. Age-matched and non-age-matched studies were analyzed in separate. In analysis of age-matched studies, 3683 MS patients and 1304 age-matched healthy controls were included (Table 2). There was obvious between-study heterogeneity (I^2^ = 65.0%) and the random-effect model was used. The blood NfL levels in MS were significantly higher than those in age-matched controls with a moderate effect size (SMD = 0.64, 95%CI 0.44–0.85, P<0.001, Fig 2). We observed large effect size in studies recruiting treatment-naïve MS patients (SMD = 0.91, 95%CI 0.39–1.43) and moderate effect size in studies with mixed use or missing data of DMT (SMD = 0.56, 95%CI 0.32–0.80; between-subgroup comparison P = 0.236). Blood NfL difference between MS and non-matched controls was analyzed in 14 studies comprising 1414 MS patients and 1375 controls (Table 2). MS patients had significantly higher NfL levels than non-matched controls (SMD = 0.76, 95%CI 0.56–0.96, P<0.001, S1 Fig). Between-subgroup comparison showed a significantly larger effect size of treatment-naïve subgroup than treatment subgroup (SMD = 1.20 vs. 0.65, P = 0.007).

**Table 2:** Summary of meta-analysis for diagnosis value of blood NfL concentrations.

Analysis	No. of studies	No. of participants	Pooled effect size			Heterogeneity	
			SMD	95%CI	P	I^2^, %	P
**MS vs. Control, age-matched**	10	3683/1304	0.64	0.44–0.85	<0.001	65.0	0.002
Control type							
HC	9	3616/1267	0.67	0.45–0.90	<0.001	66.7	0.002
NINDC	1	67/37	0.42	0.01–0.82	0.044	-	-
Sample source							
Serum	8	539/255	0.62	0.30–0.95	<0.001	72.4	<0.001
Plasma	2	3144/1049	0.69	0.62–0.77	<0.001	0	0.830
NfL detection method							
SIMOA	9	3648/1281	0.66	0.44–0.88	<0.001	68.1	0.002
ECL or ELISA	1	35/23	0.47	-0.07, 1.00	0.085	-	-
DMT use							
No	4	182/92	0.91	0.39–1.42	<0.001	71.4	0.015
Mixed or missing data	6	3501/1212	0.56	0.32–0.80	<0.001	66.6	0.010
**MS vs. Control, not matched**	14	1414/1375	0.76	0.56–0.96	<0.001	81.5	<0.001
Control type							
HC	12	1352/1315	0.74	0.53–0.95	<0.001	83.5	<0.001
NINDC	2	62/60	0.94	0.37–1.51	0.001	55.4	0.135
Sample source							
Serum	13	1255/1309	0.78	0.57–0.99	<0.001	82.6	<0.001
Plasma	1	159/66	0.54	0.25–0.83	<0.001	-	-
NfL detection method							
SIMOA	12	1283/1265	0.75	0.53–0.97	<0.001	83.8	<0.001
ECL or ELISA	2	131/110	0.87	0.61–1.14	<0.001	0	0.565
DMT use							
No	3	197/152	1.20	0.85–1.55	<0.001	50.5	0.133
Mixed or missing data	11	1217/1223	0.65	0.46–0.84	<0.001	76.5	<0.001
**RRMS vs. HC**	16	1239/858	0.58	0.36–0.80	<0.001	79.0	<0.001
**PMS vs. HC**	8	362/522	1.01	0.65–1.36	<0.001	76.1	<0.001
**MS vs. CIS**	3	672/487	0.30	0.18–0.42	<0.001	0	0.519
**PMS vs. RRMS**	10	842/419	0.56	0.27–0.85	<0.001	79.8	<0.001
**Relapse vs. Remission**	6	181/600	0.54	0.16–0.92	0.005	69.0	0.007

SIMOA: single molecular array; SMD: standard mean difference

**Fig 3:**
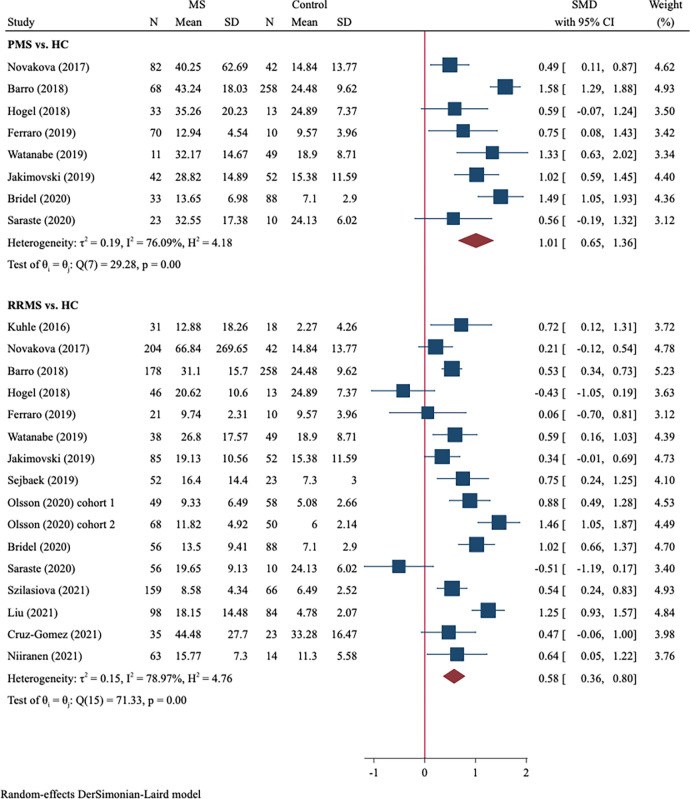
Forest plot of blood NfL concentrations between PMS *vs*. HC and RRMS *vs*. HC. PMS: progressive MS; RRMS: relapsing-remitting MS.

We further compared the blood NfL levels in patients at different MS stages (RRMS and PMS) with those in HC. A total of 1239 RRMS vs. 858 HC from 16 studies and 362 PMS vs. 522 HC from 8 studies were included. RRMS patients had significantly higher levels of blood NfL (SMD = 0.58, 95%CI 0.36–0.80, P<0001, Fig 3) compared with HC, which showed a moderate effect size. Moreover, a large effect size of the blood NfL difference between PMS patients and HC was observed (SMD = 1.01, 95%CI 0.65–1.36, P<0.001, Fig 3).

### MS vs. CIS

Three studies involving 672 MS and 487 CIS compared blood NfL levels between both groups. Among them, Disanto *et al* defined CIS according to the criteria proposed by Miller *et al* [52], and the other two according to 2010 revised McDonald criteria [20]. There was no between-study heterogeneity. Meta-analysis using the fixed-effect model was used showed significantly higher blood NfL levels in MS than in CIS (SMD = 0.30, 95%CI 0.18–0.42, P<0.001, S2 Fig).

### PMS vs. RRMS

**Fig 4:**
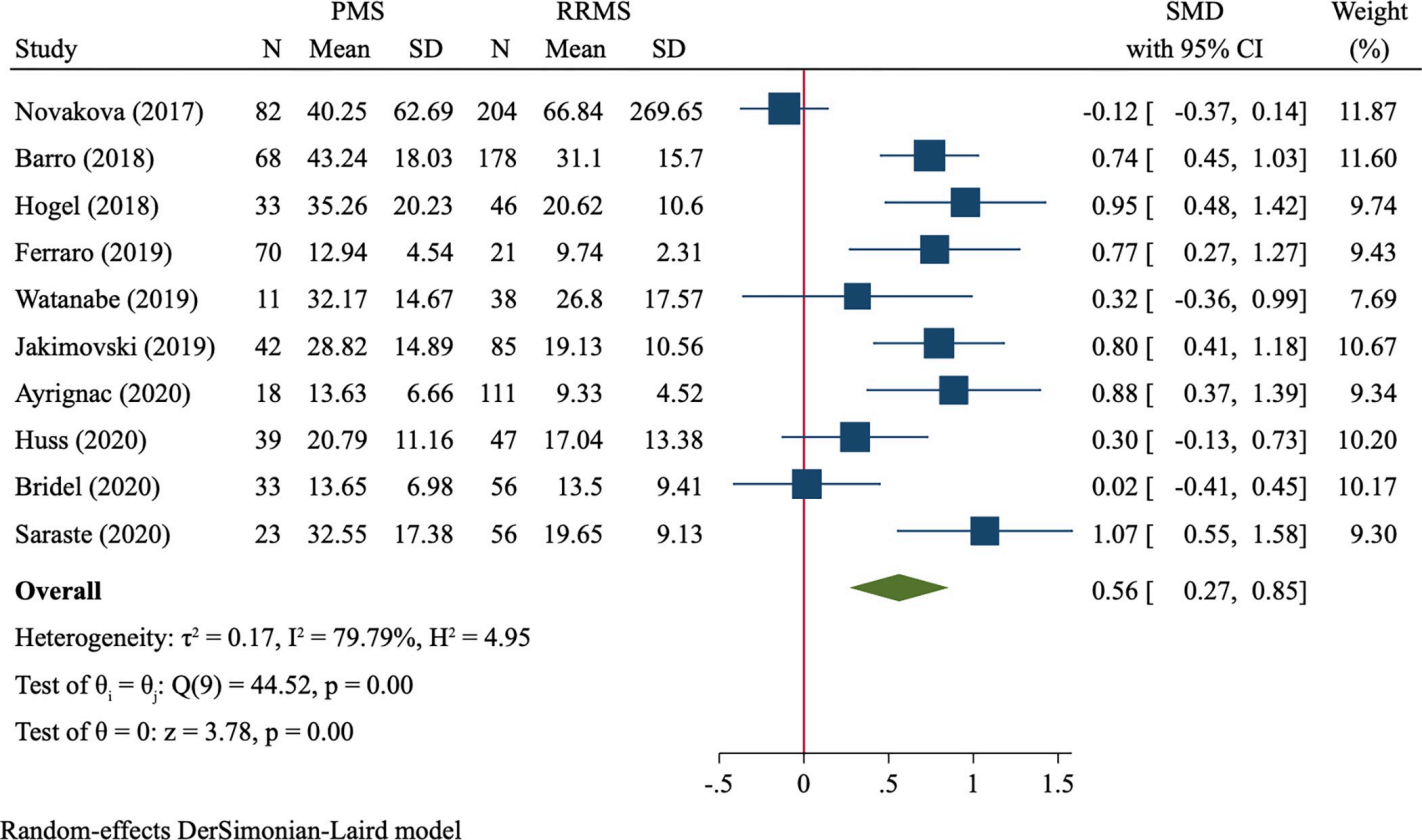
Forest plot of blood NfL levels between PMS *vs*. RRMS.

A total of 842 PMS and 419 RRMS were included, and the random-effect model was used due to substantial heterogeneity (I^2^ = 79.8%). We found that PMS patients had significantly higher levels of blood NfL than RRMS patients (SMD = 0.56, 95%CI 0.27–0.85, P<0.001, Fig 4).

### MS in relapse vs. MS in remission

**Fig 5:**
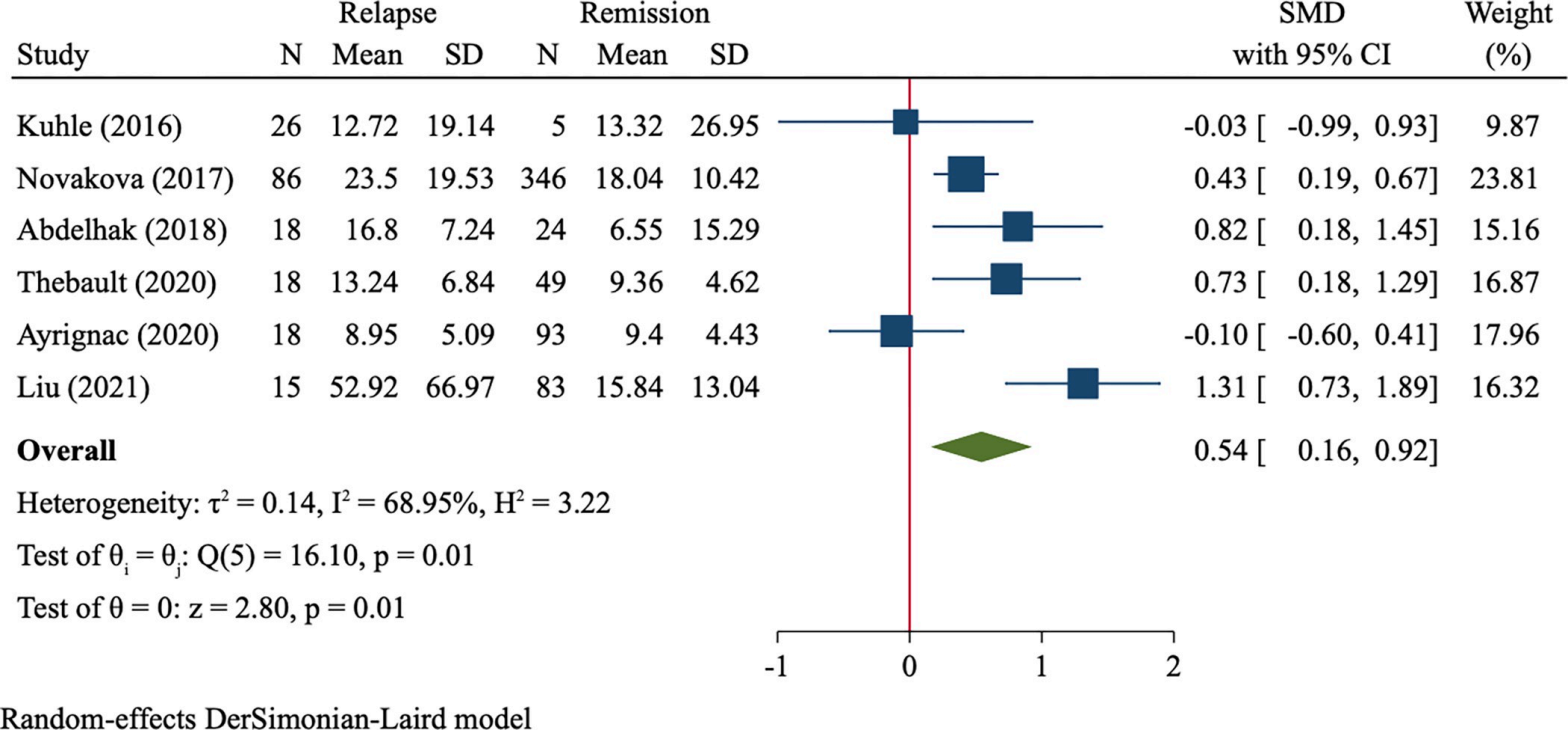
Forest plot of blood NfL levels between MS in relapse *vs*. MS in remission.

Six studies compared blood NfL levels of MS in relapse vs. MS in remission (181 cases vs. 600 cases) and were included in synthesis analysis. Random-effect model analysis demonstrated higher NfL levels in relapsed patients than in remitted patients (SMD = 0.54, 95%CI 0.16–0.92, P = 0.005, Fig 5).

### Predictive value of high blood NfL level

We investigated whether high blood NfL level at baseline could predict the hazard of reaching EDSS score≥4.0 and relapse. Patients with higher blood NfL levels were earlier to reach EDSS score≥4.0 compared with those with lower levels (HR = 2.36, 95%CI 1.32–4.21, P = 0.004, S3 Fig). However, no difference of time to relapse was observed between both groups (HR = 1.32, 95%CI 0.90–1.93, P = 0.155, S4 Fig).

### Meta-regression analysis, sensitivity analysis and publication bias

We explored the potential source of heterogeneity by meta-regression analysis in “MS vs. Control” comparison (Table 3). Mean age was significantly correlated with SMD estimates in not-age-matched subgroup (P = 0.021, S5 Fig), indicating that mean age could partly explain the source of heterogeneity. However, the correlation was not found in age-matched subgroup (P = 0.488, S6 Fig). The association of SMD with percent of female, mean EDSS score, mean disease duration and sample size were not evident according to meta-regression analysis.

**Table 3:** Results of meta-regression for blood NfL difference between MS and controls.

Covariate	Coefficient	SE	t	P
Age-matched				
Mean age	-0.014	0.02	-0.65	0.488
Percent of female	-1.01	1.28	-0.79	0.430
Mean disease duration	-0.015	0.035	-0.43	0.670
Mean EDSS score	0.148	0.131	1.13	0.259
Sample size^#^	-0.0013	0.0032	-0.39	0.696
Not age-matched				
Mean age	-0.041	0.018	-2.30	0.021
Percent of female	1.25	1.85	0.68	0.498
Mean disease duration	-0.033	0.02	-1.63	0.103
Mean EDSS score	-0.090	0.098	-0.92	0.358
Sample size	-0.0006	0.0007	-0.86	0.387

^#^ Excluding Manouchehrinia et al’s study that had a very large sample size.

Sensitivity analysis using Leave-One-Out method demonstrated that omitting one single study did not significantly influence the pooled effect size of the rest of studies. There was no obvious asymmetry in funnel plots of meta-analyses, and Egger’s test indicated no evident publication bias (S4 Table).

## Discussion

As far as we know, this is the first meta-analysis investigating the diagnostic and predictive value of blood NfL concentrations in MS patients. In line with previous meta-analyses finding elevated CSF NfL concentration in MS patients [7, 53–55], the present study demonstrates NfL levels in blood, which are strongly correlated with those in CSF, are also significantly higher in MS patients compared with controls. Our study indicates that blood NfL may serve as a biomarker for MS diagnosis.

However, some influential factors, such as age, BMI and quantification process, should be noted upon the clinical utility of blood NfL [56]. Blood NfL levels are highly age-dependent. Among healthy controls, young individuals have low and relatively stable sNfL concentrations while people older than 60 years have annually increased sNfL levels associated with age-related neurodegeneration [14, 57]. Besides, sNfL decreases with BMI in age stratified subgroups [58, 59]. Therefore, age and BMI are confounding factors for sNfL as a biomarker, which may influence the clinical implementation. The comparison between MS and unmatched controls may introduce some bias to the meta-analysis. This is supported by our meta-regression analysis revealing a negative correlation between mean age and blood NfL difference in not-age-matched studies (P = 0.021). On the contrary, among studies recruiting age-matched controls, mean age was not associated with blood NfL difference (P = 0.488). These results indicate that age-specific reference of blood NfL should be established. Recently, several studies have tried to construct an age- and/or BMI-adjusted model for sNfL [16, 51]. Using multiple large datasets, Benkert *et al* established an age- and BMI-corrected reference database of sNfL values, and further showed the merit of sNfL percentiles and Z scores in predicting disease course and response to DMT [16]. Thus, age-corrected sNfl value or a composite index may be more reliable and can be used in future researches.

Besides of age, DMT use is another influential factor of blood NfL. DMT-treated patients had significantly lower sNfL levels in untreated patients, and the treatment effect was independent of all the other baseline variables as suggested by multivariate analysis [14]. In our meta-analysis, several studies only recruited patients who had not previsouly been treated with DMT. Subgroup analyses, in both age-matched and non-matched studies, demonstrated a larger SMD effect size in treatment-naïve subgroup than treatment subgroup, suggesting a potential role of DMT in reducing blood Nfl. Follow-up of DMT-treated patients showed significantly reduced sNfL levels than baseline, which were not observed in untreated patients [30]. These observations also suggest that longitudinal sampling of blood NfL may help monitor DMT treatment effect in MS patients. However, the impact of DMT on blood NfL may vary among disease subtypes. Teriflunomide reduced sNfL in relapsing MS patients [60] and dimethyl fumarate decreased blood NfL in RRMS patients [36]. Whereas, no significant changes were observed in PMS patients treated with ibudilast [61] and SPMS patients with simvastatin treatment [62].

NfL is not a biomarker specific to MS. It reflects neuro-axonal damage and can be detected in elevated levels in the other inflammatory neurologic disorders. Despite higher blood NfL levels in MS than in NINDCs, no significant difference is observed between MS and inflammatory neurological disease controls (INDCs) [25, 30]. This phenomenon is also found in CSF measurements [8]. Both CSF and blood NfL cannot replace conventional MRI for differential diagnosis between MS and the other inflammatory neurologic disorders.

Apart from disease diagnosis, blood NfL may help differentiate MS from CIS and distinguish MS subtypes. CSF NfL can be used to distinguish CIS from healthy controls with high accuracy [63], whereas a recent meta-analysis showed no significant difference of CSF NfL levels between MS and CIS [7]. In present study, we found blood NfL levels were significantly higher in MS patients than in CIS patients. Bittner *et al* validated the application of sNfL in reclassifying CIS under McDonald diagnostic criteria 2010 (i.e. CIS[2010]) as CIS or RRMS under McDonald diagnostic criteria 2017 (i.e. CIS[2017] and RRMS[2017]), and found the inclusion of sNfL to McDonald diagnostic criteria significantly increased the area under the curve [33]. Blood NfL may be a useful biomarker for differential diagnosis between CIS and MS.

We observed higher blood NfL concentrations in PMS patients than in RRMS patients with moderate effect size. This may be attributed to greater inflammatory activity in this group of patients, especially in secondary PMS (SPMS) [56], as well as older age of PMS patients than RRMS patients. Several included studies comparing PMS and RRMS showed significantly older age and higher NfL levels of PMS patients [26, 37, 49, 50]. After correction for age, PMS still had higher sNfL levels than RRMS patients [29]. Several studies revealed that RRMS patients with higher serum NfL levels had greater risk of conversion to SPMS [32, 34, 64]. However, there is no such difference in CSF samples, and even opposite results were observed in some meta-analyses [7, 54]. In addition, we found blood NfL levels were higher in relapsed MS patients than in remitted patients, which was similar to what has been found in CSF samples [7, 54].

Blood NfL is associated with future disease activity and progression [65]. Patients with baseline higher sNfL levels had higher risk of experiencing relapse, accelerated brain and spinal cord volume loss, and EDSS worsening post blood sampling [14, 29]. Upper tertile of longitudinal measures of sNfL predicted higher risk of EDSS worsening in a long term as far as 15 years [66]. We further assessed whether blood NfL could predict time to relapse and EDSS worsening through meta-analysis. Patients with high NfL levels were earlier to reach EDSS score≥4.0 but had comparable time to relapse compared with those with low NfL levels. Thus, blood NfL can be used to predict disease progression of MS patients.

Several limitations in our study should be noted. Firstly, there was substantial between-study heterogeneity, which may be caused by cofounders such as age, gender, disease activity, and DMT usage. Secondly, the sample size of some subgroups, including PMS vs. RRMS, MS in relapse vs. MS in remission and the predictive value, was small. Thirdly, NfL levels in most studies were not in normal distribution and shown as median with IQR or range. We had to convert them into mean with SD, which did not accurately reflect the difference. Patient-level data may be warranted.

In conclusion, the present meta-analysis demonstrates that blood NfL is a potential biomarker for MS diagnosis, MS subtype differentiation, and the prediction of disease worsening.

## Supporting information

S1 Checklist (DOCX)

S1 Table Characteristics of studies included in meta-analysis for predictive value of blood NfL concentration. (DOCX)

S2 Table Quality assessment for studies included in meta-analysis of diagnosis value of blood NfL concentration according to NOS (case-control studies). (DOCX)

S3 Table Quality assessment for studies included in meta-analysis of predictive value of blood NfL concentration according to NOS (cohort studies). (DOCX)

S4 Table Egger’s test for publication bias. (DOCX)

S1 Fig Forest plot of blood NfL concentrations between MS patients *vs*. non-matched controls. (TIF)

S2 Fig Forest plot of blood NfL levels between MS patients *vs*. CIS patients. CIS: clinically isolated syndrome.
(TIF)

S3 Fig Forest plot of high blood NfL levels predicting time to reach EDSS score≥4.0. EDSS: Expanded Disability Status Scale; HR: hazard ratio.
(TIF)

S4 Fig Forest plot of high blood NfL levels predicting time to relapse. (TIF)

S5 Fig Meta-regression analysis of mean age in correlation with NfL difference between MS and non-matched controls. (TIF)

S6 Fig Meta-regression analysis of mean age in correlation with NfL difference between MS and age-matched controls. (TIF)
