# Supplementary material for: Neurofilament light chain in blood as a diagnostic and predictive biomarker for multiple sclerosis: A systematic review and meta-analysis
Source: PLoS One. 2022 Sep 14;17(9):e0274565. doi: 10.1371/journal.pone.0274565 (PMC9473405; doi:10.1371/journal.pone.0274565)
Supplement: S1 Table — (DOCX) [file pone.0274565.s002.docx]

S1 Table. Characteristics of studies included in meta-analysis for predictive value of blood NfL concentration.

| Author | Year | Sample source | NfL cutoff | Sample size | | %Female | Age, y | EDSS score | Disease duration, y | Follow-up duration | Outcome |
| --- | --- | --- | --- | --- | --- | --- | --- | --- | --- | --- | --- |
|  |  |  |  | High NfL | Low NfL |  |  |  |  |  |  |
| Lin | 2021 | Serum | ≥80th percentile of age-corrected reference values | 27 | 51 | 64.1 | 33.7 | 1.5 | 1 | 23.9 months (IQR 23.3-24.7) | Relapse |
| Haring | 2020 | Plasma | ≥30pg/mL (geometric mean) | 85 | 145 | 65.8 | 37 | 2 | 7.2 | 8.8 years (IQR 3.7-9.2) years | EDSS score≥4.0 |
| Thebault | 2020 | Serum | Tertile 3 (>13.2 pg/ml) | 22 | 22 | 70.1 | 38 | 1.5 | 15.8 | 18.9 years (range: 15.0-27.0) | EDSS score≥4.0 |
|  |  |  | Tertile 2 (7.8-13.2 pg/ml) | 22 | 22 |  |  |  |  |  |  |
| Manouchehrinia | 2020 | Plasma | ≥80th percentile of age-specific values in controls | 2299 | 1607 | NA | NA | NA | NA | 5.1 years | EDSS score≥4.0 |
| Anderson | 2020 | Serum | ≥13.7pg/mL (median) | 82 | 82 | 71 | 35.1 | 2.5 | NA | >5 years | Relapse |

NfL: neurofilament light chain; IQR: interquartile range; EDSS: Expanded Disability Status Scale; NA: not available.
