# Supplementary material for: Neurofilament light chain in blood as a diagnostic and predictive biomarker for multiple sclerosis: A systematic review and meta-analysis
Source: PLoS One. 2022 Sep 14;17(9):e0274565. doi: 10.1371/journal.pone.0274565 (PMC9473405; doi:10.1371/journal.pone.0274565)
Supplement: S2 Table — (DOCX) [file pone.0274565.s003.docx]

S2 Table. Quality assessment for studies included in meta-analysis of diagnosis value of blood NfL concentration according to NOS (case-control studies).

| Study | Selection | | | | Comparability | | Exposure | | | Total stars |
| --- | --- | --- | --- | --- | --- | --- | --- | --- | --- | --- |
|  | Item 1 | Item 2 | Item 3 | Item 4 | Item 5a | Item 5b | Item 6 | Item 7 | Item 8 |  |
| Disanto (2015) | * |  |  | * |  |  | * | * | * | 5 |
| Kuhle (2016) | * |  |  | * |  |  | * | * | * | 5 |
| Disanto (2017) | * | * |  | * |  |  | * | * | * | 6 |
| Piehl (2017) | * |  |  | * |  |  | * | * | * | 5 |
| Novakova (2017) | * |  | * | * |  |  | * | * | * | 6 |
| Barro (2018) | * |  |  | * |  |  | * | * | * | 5 |
| Hakansson (2018) | * | * |  | * | * | * | * | * | * | 8 |
| Abdelhak (2018) | * |  |  | * |  |  | * | * | * | 5 |
| Hogel (2018) | * |  |  | * | * |  | * | * | * | 6 |
| Ferraro (2019) | * |  |  | * |  |  | * | * | * | 5 |
| Watanabe (2019) | * |  |  | * |  |  | * | * | * | 5 |
| Thebault (2019) | * |  |  | * |  |  | * | * | * | 5 |
| Jakimovski (2019) | * | * | * | * | * |  | * | * | * | 8 |
| Sejbaek (2019) | * | * |  | * | * | * | * | * | * | 8 |
| Baldassari (2019) | * | * |  | * | * | * | * | * | * | 8 |
| Manouchehrinia (2020) | * | * | * | * | * | * | * | * | * | 9 |
| Bittner (2020) | * | * |  | * |  |  | * | * | * | 6 |
| Thebault (2020) | * |  |  | * | * | * | * | * | * | 7 |
| Ayrignac (2020) | * | * |  | * |  |  | * | * | * | 6 |
| Huss (2020) | * |  |  | * |  |  | * | * | * | 5 |
| Olsson (2020) | * |  |  | * |  |  | * | * | * | 5 |
| Bridel (2020) | * | * |  | * | * |  | * | * | * | 7 |
| Saraste (2020) | * |  |  | * | * |  | * | * | * | 6 |
| Szilasiova (2021) | * | * |  | * |  | * | * | * | * | 7 |
| Liu (2021) | * |  |  | * |  |  | * | * | * | 5 |
| Cruz-Gomez (2021) | * |  |  | * | * | * | * | * | * | 7 |
| Niiranen (2021) | * |  |  | * |  |  | * | * | * | 5 |
| Harp (2022) | * |  |  | * |  |  | * | * | * | 5 |

Item 1: Is the case definition adequate?

Item 2: Representativeness of the cases.

Item 3: Selection of Controls.

Item 4: Definition of Controls.

Item 5a: Matched for age.

Item 5b: Matched for gender.

Item 6: Ascertainment of exposure.

Item 7: Same method of ascertainment for cases and controls.

Item 8: Non-Response rate.

NOS: Newcastle-Ottawa Scale.
