# Supplementary material for: Neurofilament light chain in blood as a diagnostic and predictive biomarker for multiple sclerosis: A systematic review and meta-analysis
Source: PLoS One. 2022 Sep 14;17(9):e0274565. doi: 10.1371/journal.pone.0274565 (PMC9473405; doi:10.1371/journal.pone.0274565)
Supplement: S3 Table — (DOCX) [file pone.0274565.s004.docx]

S3 Table. Quality assessment for studies included in meta-analysis of predictive value of blood NfL concentration according to NOS (cohort studies).

| Study | Selection | | | | Comparability | | Outcome | | | Total stars |
| --- | --- | --- | --- | --- | --- | --- | --- | --- | --- | --- |
|  | Item 1 | Item 2 | Item 3 | Item 4 | Item 5a | Item 5b | Item 6 | Item 7 | Item 8 |  |
| Manouchehrinia (2020) | * | * | * | * |  |  | * | * | * | 7 |
| Thebault (2020) | * | * | * | * |  |  | * | * | * | 7 |
| Anderson (2020) | * | * | * | * |  |  | * | * | * | 7 |
| Haring (2020) | * | * | * | * |  |  | * | * | * | 7 |
| Lin (2021) | * | * | * | * |  |  | * | * | * | 7 |

Item 1: Representativeness of the exposed cohort.

Item 2: Selection of the non-exposed cohort.

Item 3: Ascertainment of exposure.

Item 4: Demonstration that outcome of interest was not present at start of study.

Item 5a: Matched for age.

Item 5b: Matched for gender.

Item 6: Assessment of outcome.

Item 7: Was follow-up long enough for outcomes to occur?

Item 8: Adequacy of follow-up of cohorts.

NOS: Newcastle-Ottawa Scale.
