# Supplementary material for: Neurofilament light chain in blood as a diagnostic and predictive biomarker for multiple sclerosis: A systematic review and meta-analysis
Source: PLoS One. 2022 Sep 14;17(9):e0274565. doi: 10.1371/journal.pone.0274565 (PMC9473405; doi:10.1371/journal.pone.0274565)
Supplement: S4 Table — (DOCX) [file pone.0274565.s005.docx]

S4 Table. Egger's test for publication bias.

| Analysis | Egger's test P value |
| --- | --- |
| MS vs. Control, age-matched | 0.748 |
| MS vs. Control, not matched | 0.305 |
| PMS vs. HC | 0.296 |
| RRMS vs. HC | 0.056 |
| PMS vs. RRMS | 0.378 |
| Relapse vs. Remission | 0.896 |
