# Supplementary figures and images for: Neurofilament light chain in blood as a diagnostic and predictive biomarker for multiple sclerosis: A systematic review and meta-analysis
Source: PLoS One. 2022 Sep 14;17(9):e0274565. doi: 10.1371/journal.pone.0274565 (PMC9473405; doi:10.1371/journal.pone.0274565)

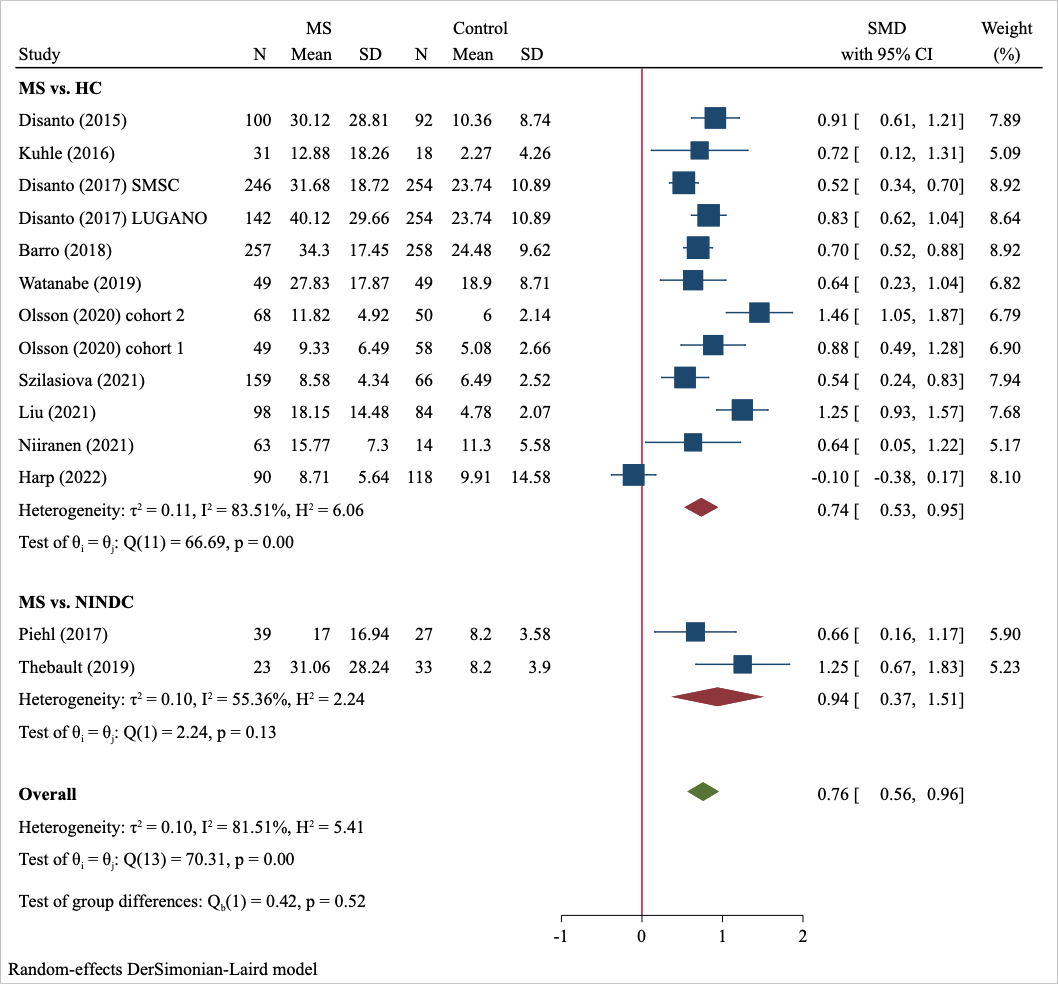

Supplement: S1 Fig — (TIF) [file pone.0274565.s006.tif]

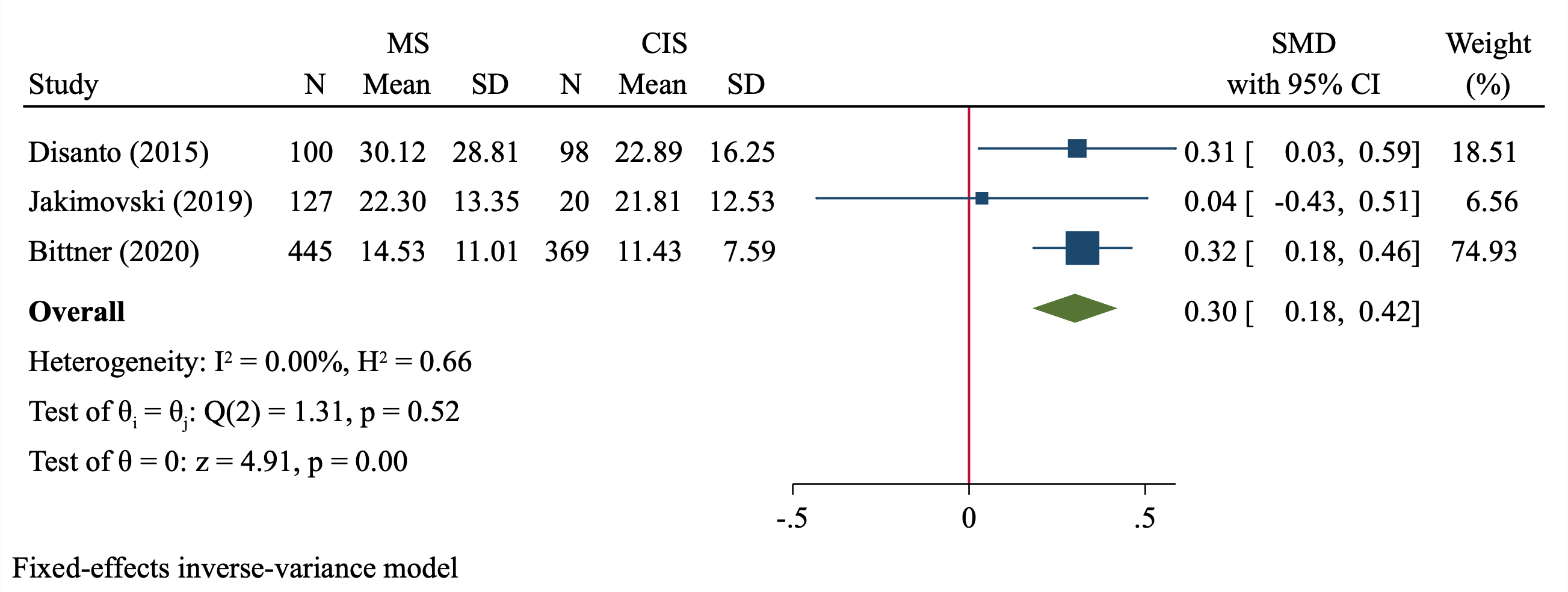

Supplement: S2 Fig — CIS: clinically isolated syndrome. (TIF) [file pone.0274565.s007.tif]

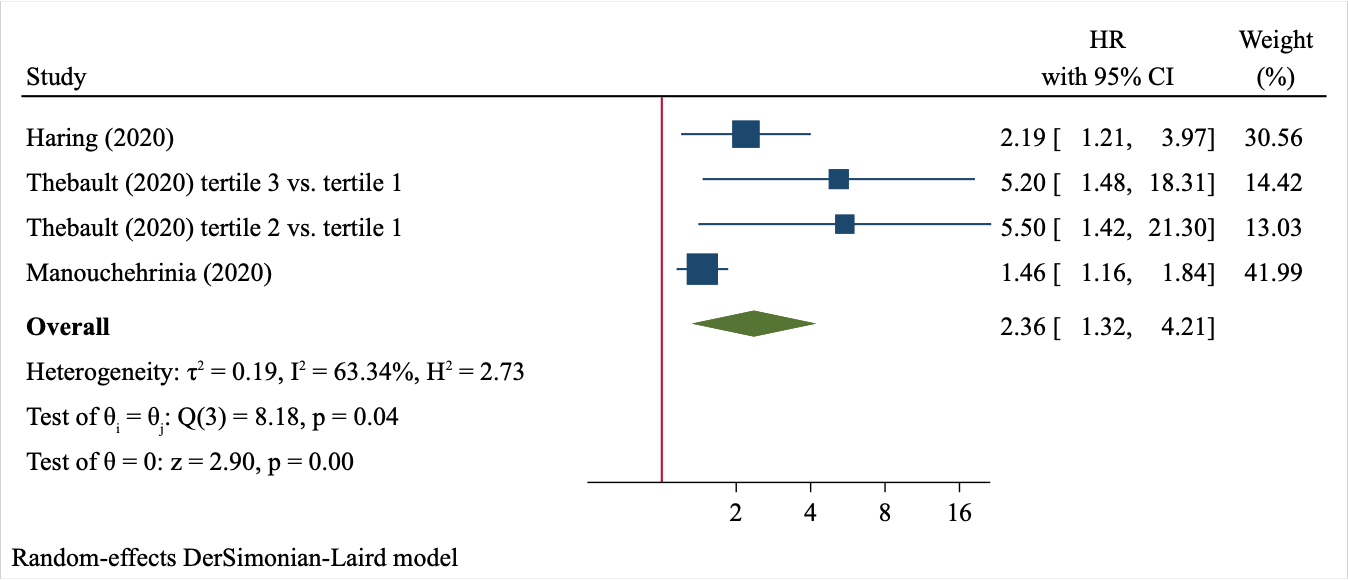

Supplement: S3 Fig — EDSS: Expanded Disability Status Scale; HR: hazard ratio. (TIF) [file pone.0274565.s008.tif]

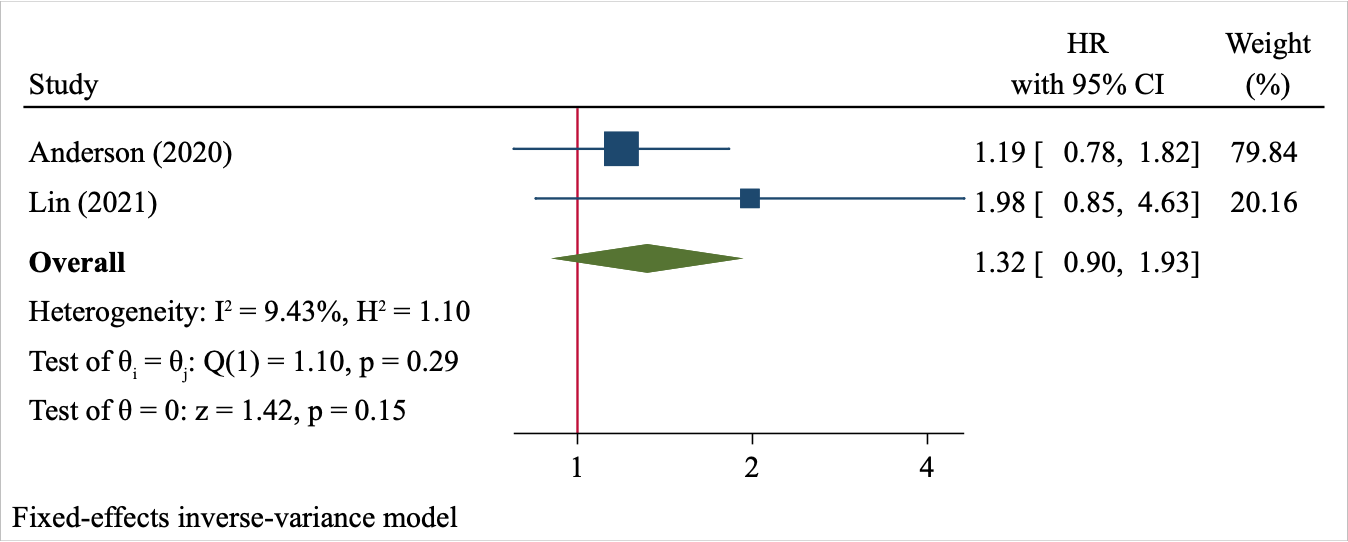

Supplement: S4 Fig — (TIF) [file pone.0274565.s009.tif]

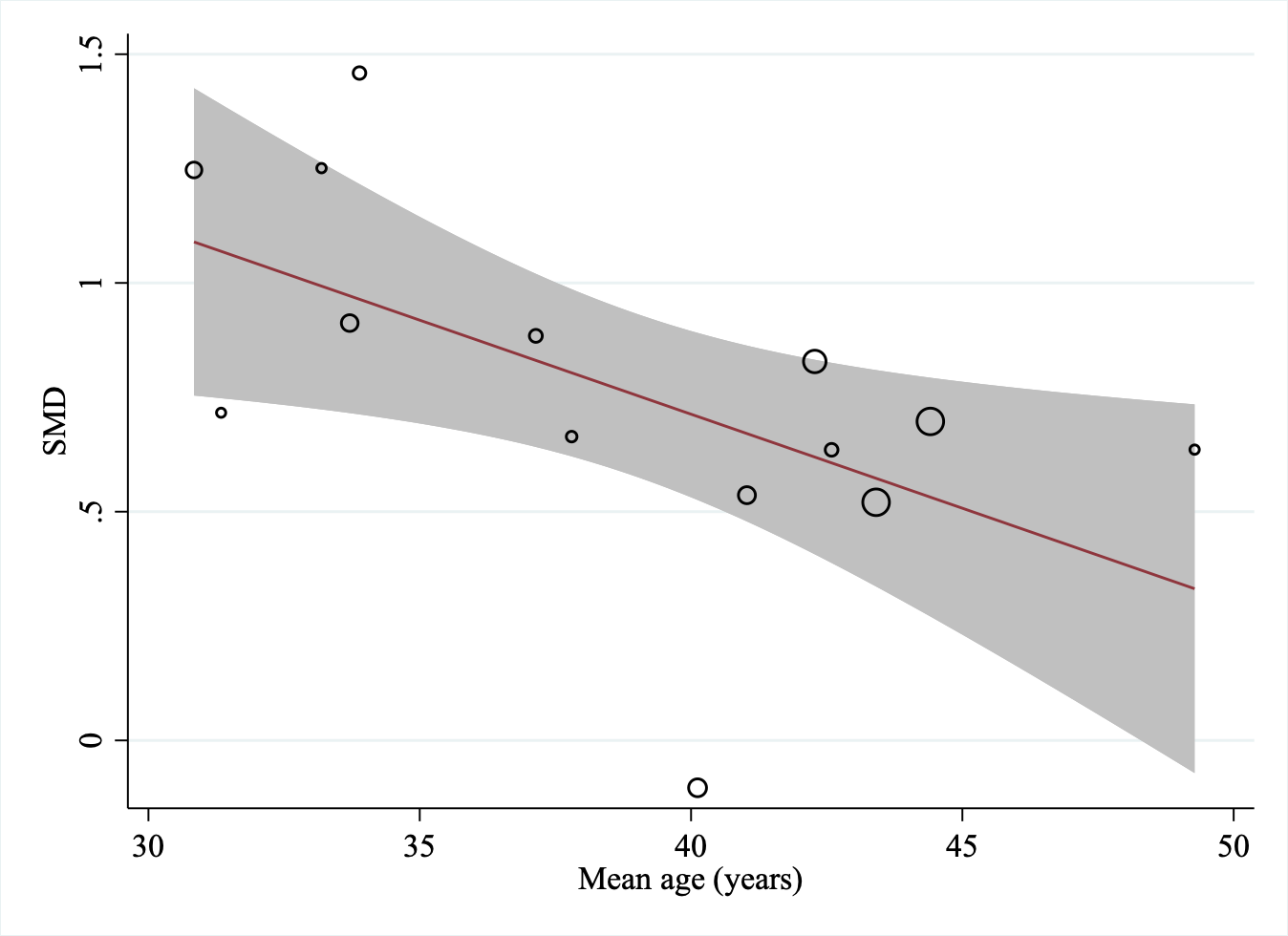

Supplement: S5 Fig — (TIF) [file pone.0274565.s010.tif]

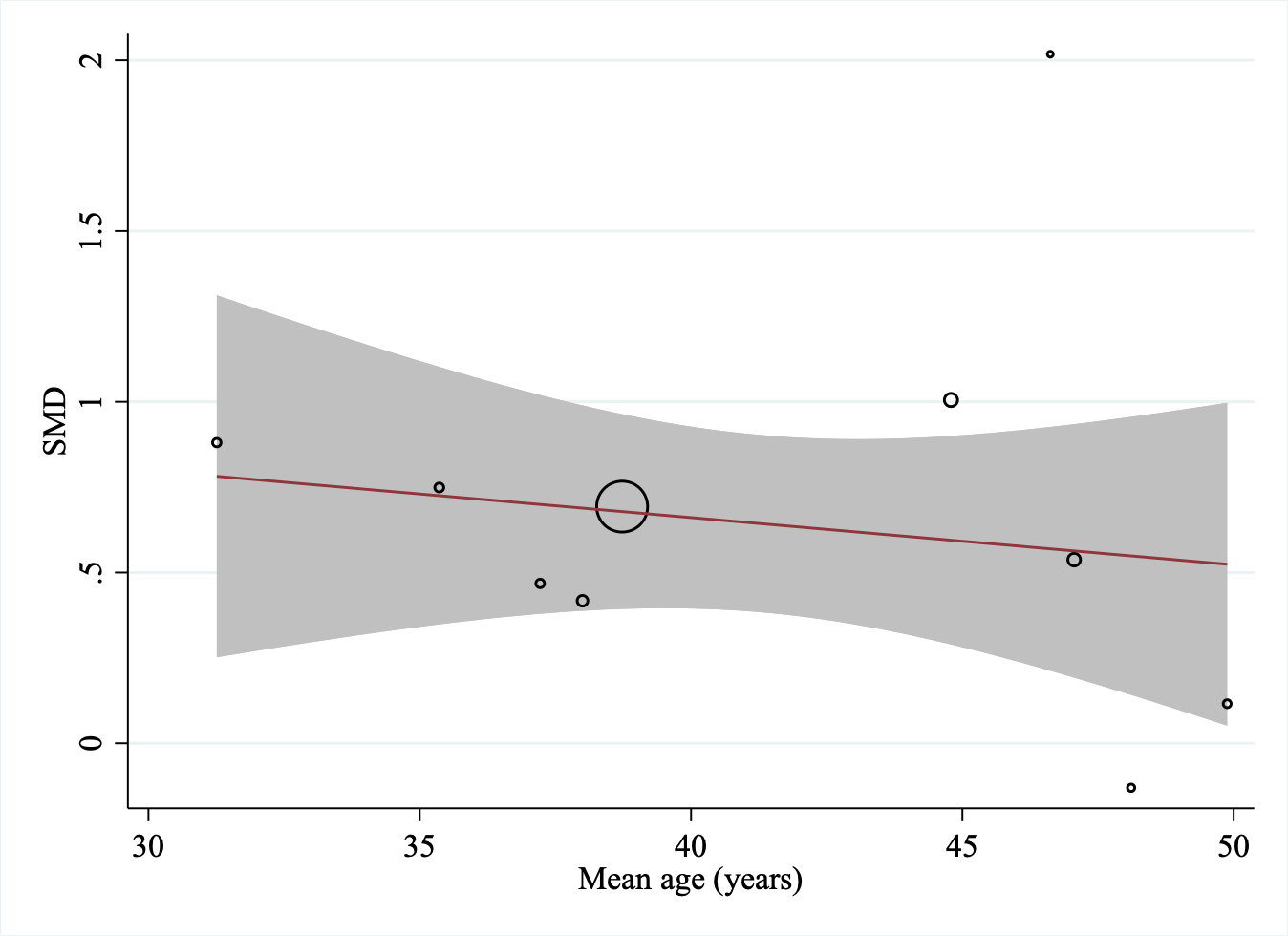

Supplement: S6 Fig — (TIF) [file pone.0274565.s011.tif]
